# Chlorido(2-formyl-6-hydroxy­phenyl-κ*C* ^1^)mercury(II)

**DOI:** 10.1107/S160053680902145X

**Published:** 2009-06-10

**Authors:** Chen Xu, Fei-Fei Cen, Zhi-Qiang Wang, Yu-Qing Zhang

**Affiliations:** a College of Chemistry and Chemical Engineering, Luoyang Normal University, Luoyang 471022, People’s Republic of China; b Chemical Engineering and Pharmaceutics School, Henan University of Science and Technology, Luoyang 471003, People’s Republic of China

## Abstract

In the planar [r.m.s. deviation 0.0265 Å] title compound, [Hg(C_7_H_5_O_2_)Cl], the Hg^II^ atom shows a typical linear coordination by a C atom of a benzene ring and a Cl atom. The benzene C atom and the aldehyde O atom chelate the Hg^II^ atom by assuming the Hg⋯O separation of 2.817 (9) Å as a weak intra­molecular coordination bonding distance. The resulting five-membered metallacycle is nearly coplanar with the benzene ring dihedral angle 2.9 (1)°]. Inter­molecular O—H⋯O hydrogen bonds are present in the crystal structure, resulting in a one-dimensional supra­molecular architecture parallel to [201].

## Related literature

For historical background and for properties of cyclo­metallated compounds, see: Dupont *et al.* (2005 ▶); Xu *et al.* (2009 ▶). For the properties of cyclo­mercurated compounds, see: Wu *et al.* (2001 ▶); Ryabov *et al.* (2003 ▶). For related structure, see: King *et al.* (2002 ▶); Zhou *et al.* (2005 ▶); Hao *et al.* (2007 ▶).
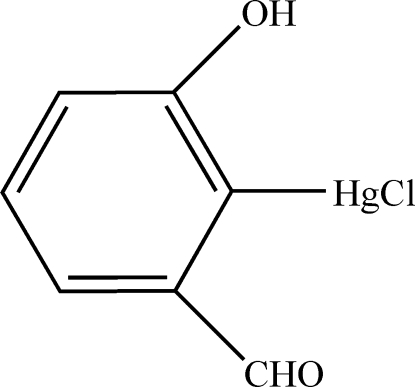


## Experimental

### 

#### Crystal data

[Hg(C_7_H_5_O_2_)Cl]
*M* *_r_* = 357.15
Monoclinic, 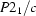

*a* = 4.7200 (19) Å
*b* = 17.702 (7) Å
*c* = 10.506 (4) Å
β = 98.839 (5)°
*V* = 867.4 (6) Å^3^
*Z* = 4
Mo *K*α radiation
μ = 18.00 mm^−1^
*T* = 296 K
0.08 × 0.01 × 0.01 mm

#### Data collection

Bruker SMART APEX CCD area-detector diffractometer
Absorption correction: multi-scan (*SADABS*; Sheldrick, 1996 ▶) *T*_min_ = 0.327, *T*_max_ = 0.841
5002 measured reflections
1595 independent reflections
1130 reflections with *I* > 2σ(*I*)
*R*_int_ = 0.050

#### Refinement

*R*[*F* ^2^ > 2σ(*F* ^2^)] = 0.045
*wR*(*F* ^2^) = 0.114
*S* = 1.01
1595 reflections
101 parameters
H-atom parameters constrained
Δρ_max_ = 0.94 e Å^−3^
Δρ_min_ = −2.32 e Å^−3^

### 

Data collection: *SMART* (Bruker, 2004 ▶); cell refinement: *SAINT* (Bruker, 2004 ▶); data reduction: *SAINT*; program(s) used to solve structure: *SHELXS97* (Sheldrick, 2008 ▶); program(s) used to refine structure: *SHELXL97* (Sheldrick, 2008 ▶); molecular graphics: *SHELXTL* (Sheldrick, 2008 ▶); software used to prepare material for publication: *SHELXTL*.

## Supplementary Material

Crystal structure: contains datablocks global, I. DOI: 10.1107/S160053680902145X/si2180sup1.cif

Structure factors: contains datablocks I. DOI: 10.1107/S160053680902145X/si2180Isup2.hkl

Additional supplementary materials:  crystallographic information; 3D view; checkCIF report

## Figures and Tables

**Table 1:** Hydrogen-bond geometry (Å, °)

*D*—H⋯*A*	*D*—H	H⋯*A*	*D*⋯*A*	*D*—H⋯*A*
O1—H1⋯O2^i^	0.82	1.93	2.730 (12)	165

Symmetry code: (i) 
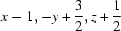
.

**Fig. 1.**
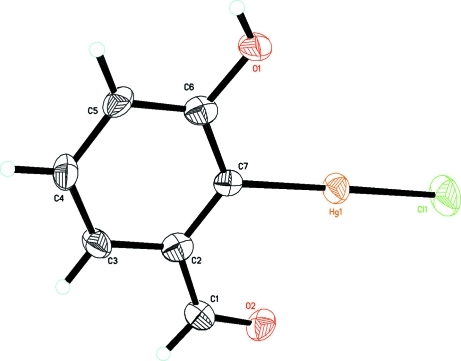
The molecular structure of the title compound with displacement ellipsoids at the 30% probability level.

**Fig. 2.**
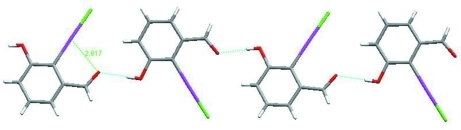
Partial view of the crystal packing showing the formation of the one-dimensional chain structure formed by the intermolecular O—H···O hydrogen bonds.

## supplementary crystallographic information

### Comment

Cyclometallated compounds containing a metal-carbon bond stabilized by the intramolecular coordination of one or two neutral atoms have a very rich chemistry and are widely used in synthesis, catalysis and materials (Dupont *et al.*, 2005; Xu *et al.*, 2009). Among them, cyclomercurated compounds are easy to prepare through a C—H activation process and their ease in undergoing transmetallation for the synthesis of other organometallic compounds (Wu *et al.*, 2001; Ryabov *et al.*, 2003).

In the planar title compound (Fig. 1), the mercury(II) atom shows a typical linear coordination geometry with a carbon atom of the benzene ring and the chloride atom in *trans* position. O2—Hg1 distance (2.817 (9) Å) is shorter than the sum of van der Waals radii (3.29 Å)of Hg and O (King *et al.*, 2002), indicating the presence of the weak intramolecular coordination, while it is longer than those of the related Hg(II) complex (Zhou *et al.*, 2005). The C—Hg and Hg—Cl bond distances are within normal ranges. The C7—Hg1—Cl1 angle is 178.1 (3)°, slightly smaller than the ideal value of 180° in organic derivatives of mercury(Hao *et al.*, 2007). Intermolecular O—H···O hydrogen bonds are present in the crystal structure (Table 1), resulting in a one-dimensional supramolecular architecture (Fig.2).

### Experimental

The title compound was prepared from the *m*-hydroxybenzaldehyde with Hg(OAc)_2_ and subsequent treatment with LiCl and recrystallized from dichloromethane-petroleum ether solution at room temperature to give the desired product as colorless crystals suitable for single-crystal X-ray diffraction (yield 82%; m.p 442–444 K). IR data (v_max/ cm^-1^): 3408, 2926, 1651, 1567, 1445, 1291, 1199, 789. NMR δ(H) 7.18(1*H*,d), 7.45 (1*H*,t), 7.52(1*H*,d), 10.12(1*H*,s), 12.11(1*H*,m).

### Refinement

H atoms attached to C atoms of the title compound were placed in geometrically idealized positions and treated as riding with C—H distances constrained to 0.93–0.96 Å, and with *U*_iso_(H)=1.2*U*_eq_(C).

### Crystal data

**Table:**

[Hg(C_7_H_5_O_2_)Cl]	*F*(000) = 640
*M**_r_* = 357.15	*D*_x_ = 2.735 Mg m^−^^3^
Monoclinic, *P*2_1_/*c*	Mo *K*α radiation, λ = 0.71073 Å
*a* = 4.7200 (19) Å	Cell parameters from 1175 reflections
*b* = 17.702 (7) Å	θ = 2.3–22.3°
*c* = 10.506 (4) Å	µ = 18.00 mm^−^^1^
β = 98.839 (5)°	*T* = 296 K
*V* = 867.4 (6) Å^3^	Block, colourless
*Z* = 4	0.08 × 0.01 × 0.01 mm

### Data collection

**Table:**

Bruker SMART APEX CCD area-detector diffractometer	1595 independent reflections
Radiation source: fine-focus sealed tube	1130 reflections with *I* > 2σ(*I*)
graphite	*R*_int_ = 0.050
φ and ω scans	θ_max_ = 25.5°, θ_min_ = 2.3°
Absorption correction: multi-scan (*SADABS*; Sheldrick, 1996)	*h* = −5→5
*T*_min_ = 0.327, *T*_max_ = 0.841	*k* = −21→21
5002 measured reflections	*l* = −12→12

### Refinement

**Table:**

Refinement on *F*^2^	Primary atom site location: structure-invariant direct methods
Least-squares matrix: full	Secondary atom site location: difference Fourier map
*R*[*F*^2^ > 2σ(*F*^2^)] = 0.045	Hydrogen site location: inferred from neighbouring sites
*wR*(*F*^2^) = 0.114	H-atom parameters constrained
*S* = 1.01	*w* = 1/[σ^2^(*F*_o_^2^) + (0.0564*P*)^2^ + 3.2348*P*] where *P* = (*F*_o_^2^ + 2*F*_c_^2^)/3
1595 reflections	(Δ/σ)_max_ = 0.001
101 parameters	Δρ_max_ = 0.94 e Å^−^^3^
0 restraints	Δρ_min_ = −2.32 e Å^−^^3^

### Special details

**Table:**

Geometry. All e.s.d.'s (except the e.s.d. in the dihedral angle between two l.s. planes)are estimated using the full covariance matrix. The cell e.s.d.'s are takeninto account individually in the estimation of e.s.d.'s in distances, anglesand torsion angles; correlations between e.s.d.'s in cell parameters are onlyused when they are defined by crystal symmetry. An approximate (isotropic)treatment of cell e.s.d.'s is used for estimating e.s.d.'s involving l.s. planes.
Refinement. Refinement of *F*^2^ against ALL reflections. The weighted *R*-factor *wR* andgoodness of fit *S* are based on *F*^2^, conventional *R*-factors *R* are basedon *F*, with *F* set to zero for negative *F*^2^. The threshold expression of*F*^2^ > σ(*F*^2^) is used only for calculating *R*-factors(gt) *etc*. and isnot relevant to the choice of reflections for refinement. *R*-factors basedon *F*^2^ are statistically about twice as large as those based on *F*, and *R*-factors based on ALL data will be even larger.

### Fractional atomic coordinates and isotropic or equivalent isotropic displacement parameters (Å^2^)

**Table:**

	*x*	*y*	*z*	*U*_iso_*/*U*_eq_	
Hg1	0.26063 (12)	0.66539 (3)	0.30597 (6)	0.0470 (2)	
O1	−0.160 (2)	0.6917 (5)	0.5036 (10)	0.050 (2)	
H1	−0.2948	0.6960	0.5439	0.074*	
O2	0.468 (2)	0.7878 (5)	0.1758 (9)	0.052 (2)	
Cl1	0.4684 (11)	0.5580 (2)	0.2416 (5)	0.0833 (14)	
C1	0.345 (3)	0.8403 (7)	0.2208 (14)	0.043 (3)	
H1A	0.3802	0.8888	0.1928	0.052*	
C2	0.151 (3)	0.8330 (7)	0.3132 (13)	0.042 (3)	
C3	0.039 (3)	0.8998 (7)	0.3574 (14)	0.051 (4)	
H3	0.0837	0.9460	0.3235	0.061*	
C4	−0.137 (3)	0.8974 (7)	0.4509 (13)	0.048 (4)	
H4	−0.2123	0.9420	0.4788	0.058*	
C5	−0.204 (3)	0.8276 (7)	0.5043 (13)	0.044 (3)	
H5	−0.3193	0.8257	0.5683	0.053*	
C6	−0.092 (3)	0.7611 (7)	0.4591 (12)	0.042 (3)	
C7	0.087 (2)	0.7635 (7)	0.3644 (11)	0.032 (3)	

### Atomic displacement parameters (Å^2^)

**Table:**

	*U*^11^	*U*^22^	*U*^33^	*U*^12^	*U*^13^	*U*^23^
Hg1	0.0477 (4)	0.0392 (3)	0.0583 (4)	0.0027 (3)	0.0213 (3)	−0.0048 (3)
O1	0.047 (6)	0.048 (5)	0.060 (6)	−0.006 (4)	0.027 (5)	0.003 (5)
O2	0.047 (6)	0.058 (6)	0.056 (6)	−0.003 (5)	0.028 (5)	−0.011 (5)
Cl1	0.098 (4)	0.044 (2)	0.117 (4)	0.016 (2)	0.045 (3)	−0.010 (2)
C1	0.033 (7)	0.043 (7)	0.054 (8)	−0.002 (6)	0.007 (6)	0.005 (7)
C2	0.029 (6)	0.053 (8)	0.043 (7)	−0.008 (6)	0.007 (6)	0.000 (6)
C3	0.058 (9)	0.034 (7)	0.070 (10)	0.012 (6)	0.038 (8)	0.012 (7)
C4	0.056 (9)	0.035 (7)	0.056 (9)	0.006 (6)	0.012 (7)	−0.010 (6)
C5	0.040 (8)	0.053 (8)	0.044 (8)	0.005 (7)	0.020 (6)	−0.007 (7)
C6	0.027 (7)	0.054 (8)	0.045 (8)	−0.012 (6)	0.007 (6)	0.000 (6)
C7	0.023 (6)	0.043 (7)	0.026 (6)	−0.004 (5)	−0.008 (5)	0.001 (5)

### Geometric parameters (Å, °)

**Table:**

Hg1—C7	2.052 (12)	C2—C3	1.403 (17)
Hg1—Cl1	2.288 (4)	C3—C4	1.382 (17)
O1—C6	1.370 (14)	C3—H3	0.9300
O1—H1	0.8200	C4—C5	1.412 (18)
O2—C1	1.229 (14)	C4—H4	0.9300
C1—C2	1.440 (18)	C5—C6	1.402 (17)
C1—H1A	0.9300	C5—H5	0.9300
C2—C7	1.394 (16)	C6—C7	1.403 (16)
			
C7—Hg1—Cl1	178.1 (3)	C3—C4—H4	119.9
C6—O1—H1	109.5	C5—C4—H4	119.9
O2—C1—C2	125.4 (12)	C6—C5—C4	118.9 (11)
O2—C1—H1A	117.3	C6—C5—H5	120.6
C2—C1—H1A	117.3	C4—C5—H5	120.6
C7—C2—C1	122.4 (12)	O1—C6—C7	117.8 (11)
C7—C2—C3	120.1 (12)	O1—C6—C5	121.2 (11)
C1—C2—C3	117.3 (11)	C7—C6—C5	121.0 (12)
C4—C3—C2	120.6 (12)	C2—C7—C6	119.2 (11)
C4—C3—H3	119.7	C2—C7—Hg1	120.8 (9)
C2—C3—H3	119.7	C6—C7—Hg1	119.9 (9)
C3—C4—C5	120.2 (11)		
			
O2—C1—C2—C7	2(2)	C3—C2—C7—C6	0.8 (19)
O2—C1—C2—C3	177.6 (14)	C1—C2—C7—Hg1	−1.9 (18)
C7—C2—C3—C4	−1(2)	C3—C2—C7—Hg1	−177.8 (10)
C1—C2—C3—C4	−176.7 (14)	O1—C6—C7—C2	177.3 (12)
C2—C3—C4—C5	1(2)	C5—C6—C7—C2	−1.1 (19)
C3—C4—C5—C6	−1(2)	O1—C6—C7—Hg1	−4.2 (16)
C4—C5—C6—O1	−177.0 (12)	C5—C6—C7—Hg1	177.4 (10)
C4—C5—C6—C7	1(2)	Cl1—Hg1—C7—C2	46 (10)
C1—C2—C7—C6	176.6 (12)	Cl1—Hg1—C7—C6	−132 (10)

### Hydrogen-bond geometry (Å, °)

**Table:**

*D*—H···*A*	*D*—H	H···*A*	*D*···*A*	*D*—H···*A*
O1—H1···O2^i^	0.82	1.93	2.730 (12)	165

Symmetry codes: (i) *x*−1, −*y*+3/2, *z*+1/2.
